# Secondary School Nutrition Policy Compliance in Ontario and Alberta, Canada: A Follow-Up Study Examining Vending Machine Data from the COMPASS Study

**DOI:** 10.3390/ijerph18073817

**Published:** 2021-04-06

**Authors:** Michelle M. Vine, Julianne Vermeer, Leonardo Romano, Daniel W. Harrington, Alexandra E. Butler, Karen A. Patte, Katelyn M. Godin, Scott T. Leatherdale

**Affiliations:** 1 School of Public Health and Health Systems, University of Waterloo, Waterloo, ON N2L 3G1, Canada; jvermeer@uwaterloo.ca (J.V.); lromano@uwaterloo.ca (L.R.); harrindw@gmail.com (D.W.H.); alle.butler@uwaterloo.ca (A.E.B.); sleatherdale@uwaterloo.ca (S.T.L.); 2 Department of Health Sciences, Brock University, St. Catharines, ON L2S 3A1, Canada; kpatte@brocku.ca; 3 Public Health Agency of Canada, Ottawa, ON K1A 0K9 ON, Canada; katelyn.m.godin@gmail.com

**Keywords:** school nutrition, school health, policy implementation, youth health, built environment

## Abstract

(1) Objective: To longitudinally assess food and beverages sold in vending machines in secondary schools (grades 9–12) participating in the COMPASS study (2015/2016 and 2018/2019) and (2) to examine if patterns and trends observed in previous years (2012/2013 to 2014/2015) are consistent with lack of policy compliance in Ontario and Alberta, Canada. (2) Methods: Policy compliance was assessed through comparing nutritional information on drink (e.g., sports drinks) and snack (e.g., chocolate bars) products in vending machines to Policy and Program Memorandum (P/PM) 150 in Ontario (required policy) and the Alberta Nutrition Guidelines for Children and Youth (recommended policy). Longitudinal results and descriptive statistics were calculated. (3) Results: Longitudinal results indicate that between Y_4_ (2015/2016) and Y_7_ (2018/2019), snack and drink vending machines remained mostly non-compliant in Ontario and Alberta, with a small proportion of Ontario drink machines changing from non-compliant to compliant. At the school level, descriptive results indicate the proportion of Ontario schools with policy-compliant snack and drink machines decreased between Y_4_ and Y_7_. Alberta schools were non-compliant for drink and snack machines. (4) Conclusions: Secondary schools continue to be non-compliant with provincial policies. School nutrition policies need to be simplified in order to make it easier for schools to be compliant. Enforcement of compliancy is also an area that deserves consideration.

## 1. Introduction

Obesity continues to be a leading public health concern, with rates increasing dramatically during adolescence and typically persisting into adulthood [1,2]. Diet is the single most important determinant of weight [3,4]. Previous research on adolescent dietary habits found that 94% of Canadian youth do not meet the recommended daily intake of fruits and vegetables [5,6], while 93% exceed their daily recommended sodium intake [7]. On average, 80% of youth consume more than one sugar-sweetened beverage per day, with 44% consuming three or more [8]. Diet quality is linked to better cognitive outcomes and academic performance [9,10] and mental health among adolescents, where higher healthy diet scores predicted higher emotional health [11].

Healthy eating is defined as eating practices and behaviours consistent with improving, maintaining, and/or enhancing health [12]. Determinants of healthy eating in children and youth include individual, i.e., knowledge, food preferences, and attitudes, and collective factors, i.e., interpersonal relations with family and peers (e.g., family meals), food availability and accessibility in the physical environment (e.g., schools, home, fast-food outlets), the social environment (e.g., television and other media advertisements), and the economic environment in which food is sold for profit [12,13,14,15].

The World Health Organization’s (WHO) framework for obesity reduction underscores the importance of continued monitoring and evaluation of school nutrition policies [16]. School-based health-promoting policies and interventions have the potential to reach all youth at school, regardless of socioeconomic status, creating equitable opportunities for impacting at-risk youth. Studies have found that many schools sell nutrient-poor foods (e.g., chocolate bars, cookies) and beverages (e.g., sugar-sweetened beverages, energy drinks) as a means of revenue generation [17,18]. A study exploring the relationship between dietary habits of students and the school food environment found that cafeteria availability and vending machines were negatively associated with healthy food choices and positively associated with unhealthy food choices [19].

School-based policies and initiatives represent potentially effective approaches for targeting health behaviours in the Canadian youth population. Given that youth between the ages of 5 and 18 consume nearly half of their daily intake of food and beverages at school [20], this environment serves as an important setting within which healthy dietary choices can be reinforced, in part, by limiting the availability and accessibility of products or items with low nutritional value. Research has suggested that environments with healthy food availability, pricing strategies, and policies regulating student nutrition may be more influential than individual power to make food choices [20].

Evidence suggests that certain school policies to improve student nutrition can be effective, leading to long-term health outcomes and behaviour change. One study found that after the implementation of a school nutrition policy in Prince Edward Island, Canada, youth consumed a greater amount of fruits and vegetables and milk and alternative products, and these behaviours were observed for five years after study conclusion [21]. In addition, another study from Canada found that grade 5 students attending schools with a healthy eating program demonstrated significantly lower levels of overweight and obesity and were more likely to report healthier diets and active lifestyles than students attending schools without programs [22].

Recent findings indicate that schools, and vending machines in particular, influence children’s diets. In a study of snack and beverage vending machine purchases in the US, 18% of middle-school students reported purchasing unhealthy snacks two or more days in the previous five days instead of purchasing lunch [23]. Another study found that in younger grades, vending machines were related—either positively or negatively—to students’ diets, depending on the items available [19]. Results from a randomized controlled trial in the Netherlands indicate that students make healthier choices when the availability of lower-calorie foods is increased in vending machines, combined with labeling and reduced prices [24]. In an Ontario-based study, findings from the Healthy Vending Machine Pilot Project demonstrate a decline in vending revenues as a result of replacing 50% of vending products with healthier snacks (e.g., yogurt, fruit, vegetables) [25]. While much research has focused on cafeteria food and beverage items, there is limited evidence regarding convenient on-campus options that are sold in vending machines, particularly from Ontario or Alberta.

### 1.1. School Nutrition Policies

The Alberta Nutrition Guidelines for Children and Youth (ANGCY) (2012) were developed for adoption in childcare, school, and recreation center settings [26]. The standards are voluntary (not required), with an objective to assist Albertans to help facilitate an environment that promotes healthy food choices and healthy attitudes about food [26]. The ANGCY encourage facilities and organizations to develop an identification system, based on the following categories: choose most often (green), choose sometimes (yellow), and choose least often (red), similar to a traffic light system. These categories are supplemented with standards related to portion size, total fiber, sugar, unhealthy fat, and sodium (salt) content per portion [26]. The standards recommend a maximum of three choices per week from the choose sometimes category. While a number of studies have evaluated the implementation of the ANGCY in recreation [27,28] and childcare facilities [29], fewer have done so in the school setting [30].

In 2010, the Ontario Ministry of Education introduced a provincial food and beverage policy (Policy and Program Memorandum: P/PM 150) that was required to be implemented in all 72 publicly funded school boards, representing ~850 schools [31]. P/PM 150 provides standards related to food and beverages in schools that are acceptable. They include sell most, which must make up at least 80% of all food (e.g., yogurt, cheese, grain-based snacks: pita chips, some breakfast cereals, some muffins, cookies and grain-based bars, some (baked) potato chips (given the complexity of the policy, please see the full Policy and Program Memorandum 150 (P/PM 150) for specific details on nutritional standards in order to ascertain in which category each snack and beverage item fits) and beverage choices (plain water, milk and hot chocolate, yogurt drinks, juices or blends, unsweetened and 100% juice); sell less, which can make up no more than 20% of all food and beverage choices, and not permitted for sale, based on Canada’s Food Guide [32]. Items not permitted for sale include items high in fat, sodium, and sugar (e.g., candy, chocolate, energy bars, licorice gum, gummies, some baked goods) and beverages (e.g., caffeinated coffee and tea, energy drinks, sports drinks, soft drinks with caffeine or ≥40 calories) [31].

A 2017 study by Vine and others [33] identified high rates of school non-compliance with P/PM 150 as it relates to food and beverages offered for sale in vending machines in the first three years of the COMPASS study (2012/2013–2014/2015 school years). The study highlighted the critical importance of monitoring and evaluating a provincially mandated school nutrition policy, as a high non-compliance with P/PM 150, specific to vending machines, was observed. Results showed that only 22% (snack vending machines) and 6% (drink vending machines) of schools exclusively offered vending machines with products that met P/PM 150 standards in 2014, and there was an observable increase in policy non-compliance over time [33]. Student-level data also indicate that compliancy of vending machines is directly related to student eating habits. Students report healthier eating habits in schools with greater compliance with P/PM 150 [33]. Findings warrant the need for further examination of compliancy patterns and trends with respect to in-school vending machines.

In an effort to communicate data about the barriers impacting the success of the policy and to strategize ways to evaluate it and provide recommendations for its improvement, Vine and others [34] convened a priority-setting workshop on the school nutrition environment in Ontario, Canada in 2017. The policy-maker (from the Ontario Ministry of Education), public health practitioners, school representatives, and health researchers engaged in a brainstorming and prioritizing exercise related to the school nutrition environment [35]. These results were shared with the policy-maker, including recommendations to improve P/PM 150 through research to support schools to become more policy-compliant by considering the role of the policy, its objective, how outcomes can be assessed, revenue sources, accountability for policy compliance, and financial implications of changing food offerings in vending machines [35].

Between 2015/2016 (Y_4_) and 2018/2019 (Y_7_) of the COMPASS study, it appears that little was done in Ontario to help schools become more compliant, for example, by providing education and/or training sessions or revising P/PM 150 to better align with implementation at the local school level. Despite this, P/PM 150 did specify that Ontario school boards were responsible for monitoring the implementation of the policy and were required to report on whether they were in full compliance at the end of the school year in which the policy was first implemented [31].

### 1.2. Research Objectives

Given the low policy compliance found in early examinations of the food and beverages for sale in Ontario and Alberta secondary school vending machines [33], a follow-up evaluation of recent data was recommended to assess patterns and trends over time. The objective of this research was to assess policy compliancy of food and beverages sold in vending machines in Ontario and Alberta secondary schools (grades 9–12) participating in the COMPASS study. The 7th wave (2018/2019) of COMPASS data was examined to determine if patterns and trends observed in wave 4 (2015/2016) are consistent with lack of compliance.

## 2. Materials and Methods

The COMPASS host study is an ongoing prospective cohort study (started in 2012/13) collecting longitudinal behavioural data from students (grades 9–12), and program, policy, and built environment data from the secondary schools those students attend in Ontario, Alberta, British Columbia, and Quebec. A full description of COMPASS and its methods is available in print [34] or online (www.compass.uwaterloo.ca; accessed on 15 January 2021). Consistent with previous research [33], the present study uses school-level built environment data collected in Ontario and Alberta schools between Year 4 (2015/16) and Year 7 (2018/2019). (Given the focus of the previous study [33] on policy compliance in Y_1_ to Y_3_ of Ontario and Alberta secondary schools within the COMPASS study, our focus is on those two provinces in this study to determine if patterns and trends observed have continued on in Y_4_ and Y_7_. As such, the current study excludes data from the provinces of Quebec and British Columbia). The present study is a follow-up to previous research [33] using observational COMPASS data specific to products available in school vending machines. A full description of COMPASS and its methods is available in print [36] or online (www.compass.uwaterloo.ca; accessed on 15 January 2021). This research and related materials received ethical approval from the University of Waterloo Office of Research Ethics and appropriate School Board Ethics committees.

### 2.1. Policy Compliance

As described elsewhere, within-school built environment data in participating COMPASS schools were collected with the Co-SEA tool [37]. Co-SEA is a direct observation tool used to collect objective data on the school food environment within schools. The tool combines audit measures from the ENDORSE environmental scan tool [38] with the additional functionality of being able to take pictures of the different within-school built environment resources identified during the audit and then storing those pictures in the corresponding data file as objective observations supporting the subjective audit measurements. We used objective data (pictures) of the school vending machines available in all of Year 4 and Year 7 COMPASS schools. Vending machines were numbered according to the first one the COMPASS team came across (1, 2, 3, etc.) during data collection in the school. Data were coded in the same way as in the previous study [33].

Policy compliancy was examined within and across years Y_4_ (2015/2016) and Y_7_ (2018/2019) by comparing nutritional information on products in vending machines to nutritional standards outlined in P/PM 150 in Ontario [31] and the Alberta Nutrition Guidelines for Children and Youth (2012) [26]. We did not include COMPASS data from Y_5_ and Y_6_, given that there were constraints on the complete data available at that time. Manual review of the nutrition facts table of each product was undertaken using publicly available online product information in order to create an inventory of vending machine items to determine in which category a product fit. If a product and its nutrition information could not be determined (e.g., photographs were blurry or missing from the database), policy compliancy was unknown.

### 2.2. Sample

Some schools participated in the study in Y_4_ and/or Y_7_. In Y_4_, there were 72 Ontario schools participating, while 9 Alberta schools participated. In Y_7_, 61 Ontario schools participated, and 8 Alberta schools participated. In total, there were 50 schools in Ontario that could be compared longitudinally, and 5 in Alberta.

In Ontario, five schools in Y_4_ and two in Y_7_ were privately funded and thus not subject to P/PM 150 standards. These schools were subsequently removed from analyses. For more information about the recruitment of COMPASS schools and school boards, please refer to reference [39] and Supplementary Materials online (https://uwaterloo.ca/compass-system/publications#technical, accessed on 15 January 2021).

### 2.3. Data Analysis

Given the unique nature of the data set, longitudinal results were calculated using SAS (9.4; SAS, Cary, NC, USA) to assess compliancy of schools over time. In doing so, the data were analyzed to find out if those who were compliant in Y_4_ remained compliant over time and to assess the change in non-compliance in Y_4_ to compliance in Y_7_ longitudinally. Schools had up to four vending machines in total, with the majority having two. Descriptive statistics were also calculated using R version 3.2.3 (for Y_4_) and SAS (9.4) in Y_7_. Data for individual snacks and drinks were aggregated to the vending machine and school level for analysis. Chi-square testing was done; however, these tests were limited due to small sample sizes (particularly in Alberta).

## 3. Results

### 3.1. Longitudinal Results

Results of the longitudinal analysis indicate that between Y_4_ and Y_7_, policy compliance of snack vending machines mostly remained the same—non-compliant (See Table 1). Of the 50 Ontario schools who stayed in the sample, 32% of vending machine 1 and 28% vending machine 2 remained non-compliant between 2015 and 2018. There was virtually no change in vending machine compliance over this time period, either from non-compliant to compliant or from compliant to non-compliant. Nearly half (44%) of vending machine 1 and 8% of vending machine 2 contained unknown snack vending items in 2015 or 2018, which impacted the extent to which we could determine products and thus compliance. Results were consistent in Alberta, with 80% of vending machine 1 remaining non-complaint over time.

At the school level, results were consistent with the vending-machine-level results that showed no change, and non-compliance was observed in 56% of school snack vending machines between 2015 and 2018 (Table 1). Results for Alberta remained consistent with those for Ontario, where 80% of schools remained non-compliant.

Results of a longitudinal analysis of drink vending machines (Table 2) were consistent with those of snack vending machines between 2015 and 2018. At the vending machine level, 14% of vending machine 1 and vending machine 3 and 10% of vending machine 2 in Ontario changed from non-compliant to compliant, indicating an improvement in policy adherence. Despite this improvement, 40% of vending machine 1 and 34% of vending machine 2 remained non-compliant over time. Results from Alberta indicate that almost all vending machines remained non-compliant over time.

At the school level, results were consistent with the vending-machine-level results for drinks; there was no change, and non-compliance was observed in 50% of school vending machines between 2015 and 2018 (Table 1). In Alberta, 80% of vending machines remained non-compliant during this time period.

### 3.2. Descriptive School-Level Results

Descriptive results at the school level were consistent with longitudinal trends (Table 1 and Table 2) from 2015/2016 and 2018/2019, indicating a decrease in the proportion of Ontario schools with policy-compliant snacks sold in vending machines between Y_4_ (30%) and Y_7_ (19%) (see Table 3). The proportion of schools with non-compliant snack vending machines increased between Y_4_ (67%) and Y_7_ (76%), indiating a relative change of +13% per year. In Alberta, most snack vending machines remained non-compliant.

Results for drinks were consistent with those for snack vending machines, indicating that the proportion of schools with policy-compliant drink machines increased between Y_4_ (21%) and Y_7_ (27%). However, non-compliancy also decreased between Y_4_ (79%) and Y_7_ (70%) (see Table 3). In Alberta, most drink vending machines were non-compliant.

### 3.3. Descriptive Vending-Machine-Level Results

At the vending-machine level (see Table 3), the proportion of policy-compliant snack vending machines decreased between Y_4_ (17%) and Y_7_ (0%) (Table 4). The opposite is true for non-compliant snack vending machines, of which the proportion increased over time (Y_4_: 78%; Y_7_: 84%). In Alberta, there was 100% policy non-compliancy at the snack vending-machine level.

Vending-level results for drink machines indicated that compliancy decreased between Y_4_ (29%) and Y_7_ (23%), while non-compliancy increased over time (Y_4_: 69%; Y_7_: 75%) (Table 4). Results in Alberta indicated that most drink vending machines were non-compliant.

## 4. Discussion

This study represents a follow-up to a recent study [33] assessing policy compliancy of secondary school vending machine contents (i.e., snacks and drinks) with Ontario’s P/PM 150 [31] and the Alberta Nutrition Guidelines for Children and Youth (2012) [26] in Alberta. Despite the mandatory school nutrition policies in Ontario, the results indicate that compliancy remains an issue, and non-compliancy is prevalent. Longitudinal results indicate that while there was an increase in compliance in drink vending machines between Y_4_ and Y_7_ (14% in vending machine 1; Table 2), many schools remained non-compliant in their snack vending machines between Y_4_ and Y_7_ (32% of vending machine 1; 28% of vending machine 2), in addition to showing a little change in compliance in either direction (change from non-compliance to compliance or vice versa). The longitudinal results also indicate that while 14% of vending machine 1 and vending machine 3 in Ontario changed from non-compliant to compliant, 40% of vending machine 1 remained non-compliant between Y_4_ and Y_7_. The descriptive results from Ontario indicate less compliancy and more non-compliancy in snack machines and more compliancy and less non-compliancy in drink machines.

The longitudinal results from Alberta are worse than those from Ontario and indicate almost complete non-compliance of snack and drink vending machines between 2015/2016 and 2018/2019. These results may, at least partially, reflect the voluntary nature of Alberta’s policy. This finding highlights the need for a mandatory school nutrition policy, including enforcement and monitoring by the Government of Alberta. Training and education to support school-level implementation is a key component of enforcement and would help schools to more successfully and confidentially implement the policy.

### 4.1. Limitations

While there are a number of strengths and contributions associated with this study, three main limitations exist. First, while the Ontario sample of COMPASS includes a significant number of publicly funded schools of all socioeconomic and geographic backgrounds, the Alberta sample is small and largely rural and therefore not representative. While not representative, it does serve as an important indicator of implementation trends in Alberta and an interesting point of comparison between the provinces of Ontario and Alberta. Second, it may be more likely that the 22 schools that dropped out of the COMPASS study over time (between Y_4_ and Y_7_) could be the ones that were not compliant, and the ones that remain could be more likely to be doing better in the area of youth health. Finally, given that P/PM 150 includes food and beverages for sale in school food outlets (e.g., cafeterias and tuck shops), these results do not reflect all of the factors that help to support or hinder the implementation of the P/PM 150.

### 4.2. Implications

These results have implications for future research and practice.

Research: Our findings provide an indication that secondary schools in both Ontario and Alberta have continued to be non-compliant with provincial nutrition policies in the context of vending machine sales. Non-compliancy was demonstrated by a high proportion of vending machine beverages containing caffeine (e.g., Diet Coke™) and/or sugar (e.g., Coca Cola™). Caffeinated beverages were commonly sold, indicating that P/PM 150 standards may not be fully articulating that caffeinated beverages are considered restricted. Policy-restricted vending foods that were being sold contained high levels of fat, sugar, and sodium, including, among other items, chips, chocolate bars, and cookies. To add to the potential uncertainty faced by policy implementers, it was often difficult, as researchers trained in nutrition and public health, to ascertain the compliancy of a brand of chip. Future research to collect sales data to provide an in-depth examination of item sales, in addition to developing and piloting more streamlined nutrition standards, is needed. At the school level, it would be valuable to provide a list of policy-compliant brands and their associated flavours in order to support successful policy implementation. There is also a need to explore the impact of P/PM 150 standards on student behaviours (e.g., leaving to buy lunch off-campus, buying lunch at school, bringing lunch from home) [40].

This research comes at an opportune time, aligning with the Ontario Ministry of Education’s desire to revise P/PM 150 nutrition standards [31]. Results of a recent Canadian Institute of Health Research-funded planning meeting on school nutrition policy include a need to ensure that a revised P/PM 150 reflects an attempt to incorporate a multi-cultural lens, involving students in the consultation process and harmonizing P/PM 150 with other school standards (e.g., menu planning, student nutrition programs) [34]. There is an urgent need for a consistent enforcement of policy and a monitoring and evaluation process for P/PM 150 in schools. Comprehensive teacher and administrator training to facilitate successful policy implementation and student capacity building could help support students and provide them with the skills and confidence to make healthy choices [34,35].

Practice: Evidence indicates that trends in policy compliancy are not changing over time, and there is a need for policies to be more context-specific in order to improve implementation at the local level [33]. Recent research illustrates the positive influence of active family and community involvement in policy development and implementation [41]. The involvement of key stakeholders (e.g., students, public health dietitians, teachers, school administrators, policy-makers, food distributors, cafeteria staff, etc.) in the design and implementation of nutrition standards and related programs may support higher P/PM 150 compliancy. The enforcement of nutrition policies in schools also requires exploration, given that there is often no enforcement of policies, despite them being mandatory. In a recent review of the implementation of school nutrition policies, McIsaac, Spencer, Chiasson, Kontak and Kirk [42] identified areas to support policy action, including the need to develop a common purpose and responsibility among stakeholders, aligning nutrition and core school priorities and addressing financial implications of healthy food access. Given the relationship between mandatory provincial school nutrition policies and more limited sugar-sweetened beverage availability in school vending machines, the findings of Godin, Hammond, Chaurasia and Leatherdale [43] provide support for mandatory school nutrition policies, including the need for enforcement of these policies.

## 5. Conclusions

School nutrition policies aim to rectify unhealthy foods sales at school and their associated consequences [44,45]. However, many barriers to their implementation exist. Even with 100% policy compliancy, vending machines are one small component of an overall comprehensive systems approach that is required to change eating behaviours. Food is complex, and a number of factors surrounding schools may limit the effectiveness of school nutrition policies in supporting healthy eating. This research raises several questions, including our need to understand how a policy that is required by a provincial government can be ignored to this extent, and the conditions that permit this situation require closer scrutiny. Relatedly, the role and responsibility of school boards and policy-makers in enforcing and monitoring nutrition policies at the school level require further consideration.

## Figures and Tables

**Table 1:** Policy compliance over time for snack vending machines (VM) in Ontario and Alberta (2015/2016–2018/2019).

VM or School Level	Change from Non-Compliance to Compliance n (%)	Change from Compliance to Non-Compliance n (%)	No Change—Stayed Compliant n (%)	No Change—Stayed Non-Compliant n (%)	Either 2015 or 2018 Contains Unknown ^a^ Vending Items n (%)	Schools with No 2015 Vending Machines n (%)	Schools with No 2018 Vending Machines n (%)	Schools with No 2015 or 2018 Vending Machines n (%)	Total n (%)
**Ontario**									
**VM 1**	0 (0)	0 (0)	0 (0)	16 (32)	22 (44)	3 (6)	1 (2)	8 (16)	50 (100)
**VM 2**	0 (0)	2 (4)	0 (0)	14 (28)	4 (8)	3 (6)	8 (16)	19 (38)	50 (100)
**VM 3**	0 (0)	0 (0)	0 (0)	1 (2)	2 (4)	2 (4)	7 (14)	38 (76)	50 (100)
**VM 4**	0 (0)	0 (0)	0 (0)	1 (2)	1 (2)	0 (0)	3 (6)	45 (90)	50 (100)
**School**	0 (0)	0 (0)	0 (0)	28 (56)	10 (20)	3 (6)	1 (2)	8 (16)	50 (100)
**Alberta**									
**VM 1**	0 (0)	0 (0)	0 (0)	4 (80)	0 (0)	1 (20)	0 (0)	0 (0)	5 (100)
**VM 2**	0 (0)	0 (0)	0 (0)	2 (40)	0 (0)	0 (0)	1 (20)	2 (40)	5 (100)
**VM 3**	0 (0)	0 (0)	0 (0)	0 (0)	0 (0)	0 (0)	1 (20)	4 (80)	5 (100)
**School**	0 (0)	0 (0)	0 (0)	4 (80)	0 (0)	0 (0)	1 (20)	0 (0)	5 (100)

^a^ Unknown schools had no non-permitted products, but the proportion of products in other categories (e.g., “Sell 80” or “Sell Most”) could not be determined with any certainty.

**Table 2:** Policy compliance over time for drink vending machines in Ontario and Alberta (2015/2016–2018/2019).

VM or School Level	Change from Non-Compliance to Compliance n (%)	Change from Compliance to Non-Compliance n (%)	No Change—Stayed Compliant n (%)	No Change—Stayed Non-Compliant n (%)	Either 2015 or 2018 Contains Unknown ^a^ Vending Items n (%)	Schools with No 2015 Vending Machines n (%)	Schools with No 2018 Vending Machines n (%)	Schools with No 2015 or 2018 Vending Machines n (%)	Total n (%)
**Ontario**									
**VM 1**	7 (14)	1 (2)	0 (0)	20 (40)	18 (36)	1 (2)	0 (0)	3 (6)	50 (100)
**VM 2**	5 (10)	2 (4)	0 (0)	17 (34)	10 (20)	2 (4)	3 (6)	11 (22)	50 (100)
**VM 3**	7 (14)	0 (0)	0 (0)	0 (0)	9 (18)	2 (4)	7 (14)	25 (50)	50 (100)
**VM 4**	0 (0)	1 (2)	0 (0)	2 (4)	3 (6)	1 (2)	3 (6)	40 (80)	50 (100)
**VM 5**	1 (2)	0 (0)	1 (2)	1 (2)	1 (2)	0 (0)	2 (4)	44 (88)	50 (100)
**School**	5 (10)	0 (0)	0 (0)	25 (50)	16 (32)	1 (2)	3 (6)	0 (0)	50 (100)
**Alberta**									
**VM 1**	0 (0)	0 (0)	0 (0)	5 (100)	0 (0)	0 (0)	0 (0)	0 (0)	5 (100)
**VM 2**	0 (0)	0 (0)	0 (0)	5 (100)	0 (0)	0 (0)	0 (0)	0 (0)	5 (100)
**VM 3**	0 (0)	0 (0)	0 (0)	3 (60)	0 (0)	0 (0)	0 (0)	2 (40)	5 (100)
**VM 4**	0 (0)	0 (0)	0 (0)	1 (20)	0 (0)	1 (20)	0 (0)	3 (60)	5 (100)
**School**	0 (0)	0 (0)	0 (0)	4 (80)	0 (0)	0 (0)	1 (20)	0 (0)	5 (100)

^a^ Unknown schools had no non-permitted products, but the proportion of products in other categories (e.g., “Sell 80” or “Sell Most”) could not be determined with any certainty.

**Table 3:** Policy-compliant schools (all snacks and drinks sold in vending machines).

	Ontario						Alberta					
	Compliant n (%)	% Relative Change (Per Year)	Non-Compliant n (%)	% Relative Change (Per Year)	Unknown ^a^ n (%)	Chi-Square Test (*p*-Value)	Compliant n (%)	% Relative Change (Per Year)	Non-Compliant n (%)	% Relative Change (Per Year)	Unknown n (%)	Chi-Square Test (*p*-Value)
**Snacks**												
**2015/2016**	20 (30)	--	45 (67)	--	2 (3)	--	2 (22)	--	7 (78)	--	0 (0)	--
**2018/2019**	12 (19)	−37%	47 (76)	+13%	3 (5)	0.1850	0 (0)	−100% *	8 (100)	XX	0 (0)	**
**Drinks**												
**2015/2016**	14 (21)	--	53 (79)	--	0 (0)	--	0 (0)	--	9 (100)	--	0 (0)	--
**2018/2019**	17 (27)	+29%	43 (70)	−11%	2 (3)	0.3300	0 (0)	0%	8 (100)	0%	0 (0)	**

^a^ Unknown schools had no non-permitted products, but the proportion of products in other categories (e.g., “Sell 80” or “Sell Most”) could not be determined with any certainty. * 100% relative decrease in compliance was due to no schools being compliant in 2018–19. ** Unable to complete chi-square test due to insufficient sample size.

**Table 4:** Policy-compliant vending machines (snacks and drinks).

	Ontario						Alberta					
	Compliant n (%)	% Relative Change (Per Year)	Non-Compliant n (%)	% Relative Change (Per Year)	Unknown ^a^ n (%)	Chi-Square Test (*p*-Value)	Compliant n (%)	% Relative Change (Per Year)	Non-Compliant n (%)	% Relative Change (Per Year)	Unknown n (%)	Chi-Square Test (*p*-Value)
**Snacks**												
**2015/2016**	14 (17)	+41%	64 (78)	−9%	4 (5)	--	0 (0)	0%	12 (100)	0%	0 (0)	--
**2018/2019**	0 (0)	−100% *	71 (84)	+8%	14 (16)	**	--	0%	13 (100)	0%	0 (0)	**
**Drinks**												
**2015/2016**	47 (29)	−6%	111 (69)	0%	4 (2)	--	1 (5)	0%	20 (95)	0%	0 (0)	--
**2018/2019**	30 (23)	−21%	100 (75)	+9%	3 (2)	0.2301	0 (0)	−100%	28 (97)	+2%	1 (3)	**

^a^ Unknown vending machines had no non-permitted products, but the proportion of products in other categories (e.g., “Sell 80” or “Sell Most”) could not be determined with any certainty. * 100% relative decrease in compliance was due to no schools being compliant in 2018–19. ** Unable to complete chi-square test due to insufficient sample size.

## Data Availability

COMPASS study data are available upon request through completion and approval of an online form: https://uwaterloo.ca/compass-system/information-researchers/data-usage-application (accessed on 1 February 2021). The datasets used during the current study are available from the corresponding author on reasonable request.

## Supplementary Materials

For more information about the recruitment of COMPASS schools and school boards are available online at: (https://uwaterloo.ca/compass-system/publications#technical; accessed on 15 January 2021).
